# In Vitro Fumonisin Biosynthesis and Genetic Structure of *Fusarium verticillioides* Strains from Five Mediterranean Countries

**DOI:** 10.3390/microorganisms8020241

**Published:** 2020-02-11

**Authors:** Giovanni Beccari, Łukasz Stępień, Andrea Onofri, Veronica M. T. Lattanzio, Biancamaria Ciasca, Sally I. Abd-El Fatah, Francesco Valente, Monika Urbaniak, Lorenzo Covarelli

**Affiliations:** 1 Department of Agricultural, Food and Environmental Sciences, University of Perugia, 06121 Perugia, Italy; giovanni.beccari@unipg.it (G.B.); andrea.onofri@unipg.it (A.O.); fv220@exter.ac.uk (F.V.); 2 Department of Pathogen Genetics and Plant Resistance, Institute of Plant Genetics, Polish Academy of Sciences, 60-479 Poznan, Poland; lste@igr.poznan.pl (Ł.S.); murb@igr.poznan.pl (M.U.); 3 National Research Council of Italy, Institute of Sciences of Food Production (ISPA-CNR), 70126 Bari, Italy; veronica.lattanzio@ispa.cnr.it (V.M.T.L.); biancamaria.ciasca@ispa.cnr.it (B.C.); 4 Food Toxins and Contaminants Department, National Research Centre, Cairo 12622, Egypt; simaged@yahoo.com

**Keywords:** *Fusarium*, ear rot, maize, fumonisins, *FUM1*

## Abstract

Investigating the in vitro fumonisin biosynthesis and the genetic structure of *Fusarium verticillioides* populations can provide important insights into the relationships between strains originating from various world regions. In this study, 90 *F. verticillioides* strains isolated from maize in five Mediterranean countries (Italy, Spain, Tunisia, Egypt and Iran) were analyzed to investigate their ability to in vitro biosynthesize fumonisin B_1_, fumonisin B_2_ and fumonisin B_3_ and to characterize their genetic profile. In general, 80% of the analyzed strains were able to biosynthesize fumonisins (range 0.03–69.84 μg/g). Populations from Italy, Spain, Tunisia and Iran showed a similar percentage of fumonisin producing strains (>90%); conversely, the Egyptian population showed a lower level of producing strains (46%). Significant differences in fumonisin biosynthesis were detected among strains isolated in the same country and among strains isolated from different countries. A portion of the divergent *FUM1* gene and of intergenic regions *FUM6*-*FUM7* and *FUM7*-*FUM8* were sequenced to evaluate strain diversity among populations. A high level of genetic uniformity inside the populations analyzed was detected. Apparently, neither geographical origin nor fumonisin production ability were correlated to the genetic diversity of the strain set. However, four strains from Egypt differed from the remaining strains.

## 1. Introduction

*Fusarium verticillioides* (Sacc.) Nirenberg is a member of the *Gibberella fujikuroi* species complex, also called *Fusarium fujikuroi* species complex (FFSC), a group of 40 closely related *Fusarium* species defined by morphological traits, sexual compatibility and DNA-based phylogenetic analysis [1,2].

In particular, *F. verticillioides* belongs to the “African” clade of the FFSC [3], and it is the main causal agent of *Fusarium* ear rot of maize (*Zea mays* L.) [4,5]. This fungus has been reported worldwide and, in particular, it prevails in drier and warmer climatic regions [6,7] such as those present in temperate, semitropical and tropical regions including European [4], Mediterranean [8], African [9] and Middle Eastern [10] maize-growing areas. For example, *F. verticillioides* was the species isolated more frequently from maize kernels harvested in Italy [11,12,13], Spain [14,15,16], Egypt [17,18,19,20,21] and Iran [22]. This is also one of the species able to biosynthesize the secondary metabolites fumonisins [23]. Specifically, *F. verticillioides* is considered the main fumonisin producer; therefore, this is the most important species associated with fumonisin contamination of maize grains [24]. Fumonisins occur worldwide in maize, including Mediterranean [4,8,24,25] farming areas, where this is one of the most widely cultivated crops [26,27]. Fumonisin accumulation in maize grains can occur in the field, following preharvest infections, and possibly continue during grain storage [28].

Contaminations strongly impair maize grain quality because of the negative impact on animal and human health [29]. Fumonisin mycotoxins can be divided into four main groups, with the most abundant fumonisins found in nature included in the B group: fumonisin B_1_ (FB_1_), fumonisin B_2_ (FB_2_) and fumonisin B_3_ (FB_3_). Among B analogues, FB_1_ is the most detected fumonisin in maize as well as the most toxicologically active [24,30]. In fact, after ingestion, fumonisins may cause a wide range of toxic effects, especially towards liver and kidneys [31,32,33,34,35]. For this reason, the European Commission has established maximum limits for the sum of FB_1_ and FB_2_ in maize for human consumption [36,37].

The amount of fumonisins found in maize kernels is also dependent on the toxigenic ability of the *F. verticillioides* populations occurring in a certain cultivated field or in a specific geographic area [38]. In fact, within the *F. verticillioides* species, fumonisin production commonly varies quantitatively because of the different strain abilities to biosynthesize different levels of these mycotoxins [15,24,39,40,41]. The amount of fumonisins produced may also vary in quantity depending on substrate [42], biotic and abiotic factors [43] as well as on the relative expression of the genes involved in the biosynthetic pathway [44]. In fact, fumonisin production in *F. verticillioides* is regulated by the *FUM* biosynthetic gene cluster [45], and some of the differences between strains can be explained by *FUM* gene sequence differences [46,47]. Thus, it is very important to determine the variations of fumonisin production by *F. verticillioides* to understand the biosynthetic potential of a certain population in a specific cultivation area.

The characterization of fumonisin biosynthesis by *F. verticillioides* strains isolated from different geographic areas has been often coupled to the study of the genetic structure of these populations to investigate the degree of genetic diversity between the different strains within the same species [44,48,49,50]. This can provide an important insight on the relationships, the variations and/or the similarities among strains originating from various regions as well as on the possible correlations between genetic variability and different fumonisin production [38,51,52,53,54]. Analyses of fumonisin biosynthesis and/or molecular characterization of *F. verticillioides* strains have been conducted in populations from different countries such as Argentina [55], Brazil [38,41,44,49], Italy [50], Iran [22,52], Ethiopia [53] and Nigeria [54].

A similar approach was adopted in the present work to characterize selected *F. verticillioides* strains originating from five Mediterranean countries to simultaneously compare them in a wider geographical context by evaluating their in vitro fumonisin production and genetic profile. Specifically, the main objectives of the present study were to:

(i) investigate the abilities of selected *F. verticillioides* strains isolated from maize kernels in five Mediterranean countries to in vitro biosynthesize FB_1_, FB_2_ and FB_3_;
(ii) characterize the genetic structure of these selected strains to assess for possible variability within strains originating from each of the surveyed countries and between the strains originating from different countries.

## 2. Materials and Methods

### 2.1. Fungal Strains

**Figure 1:**
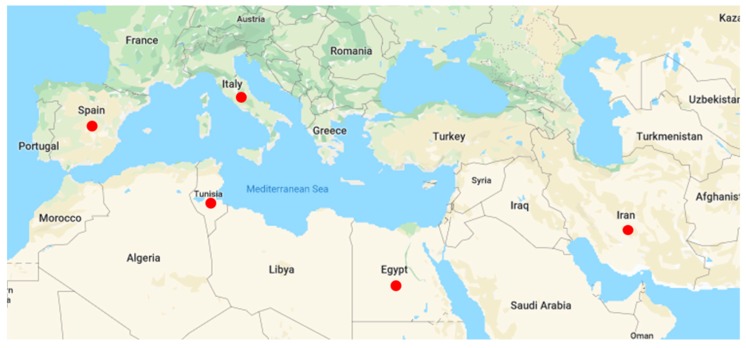
Countries of origin (red dots) of the *Fusarium verticillioides* strains used in this study. Map downloaded from www.google.com/maps and modified by the authors.

A total of 90 *F. verticillioides* strains (Table 1) isolated from single maize kernels harvested from different fields in five Mediterranean countries (22 from Italy, 9 from Spain, 16 from Tunisia, 28 from Egypt and 15 from Iran) were used in this study (Figure 1). Isolation operations were carried out in the country of origin where all strains were properly stored in fungal collections. The investigated strains had not been extensively subcultured, thus avoiding possible alterations in fumonisin production. Some of the Italian strains used in this work had been already investigated in a previous study [50] and were included to further characterize them in a wider geographical context (Figure 1).

### 2.2. Confirmation of F. verticillioides Identity by PCR Assays

To preliminarily confirm the identity of the 90 *F. verticillioides* strains used in this study, species-specific PCR assays were conducted. All strains were grown on Potato Dextrose Agar (PDA (Biolife Italiana, Milan, Italy)) at 22 °C for 14 d in the dark. DNA was extracted as described by Beccari et al. [56,57]. PCR assays were carried out with the specific primers VERT1 (GTCAGAATCCATGCCAGAACG) and VERT2 (CACCCGCAGCAATCCATCAG) [58]. A single PCR protocol was optimized using a total reaction of 20 μL. Each reaction contained 9.2 μL of sterile water for molecular biology (5prime, Hilden, Germany), 1.5 μL of cresol red (Sigma-Aldrich, Saint Louis, MO, USA), 2 μL of 10X PCR buffer (Microtech, Pozzuoli, Naples, Italy), 1.2 μL of magnesium chloride (Microtech), 2 μL of 10 mM DNTP mix (Microtech), 1 μL of 10 μM forward and reverse primers, 0.1 μL of 5 U/μL Taq polymerase (Microtech) and 2 μL of template DNA. The PCR cycle consisted of an initial denaturation step at 94 °C for 2 min, followed by 30 cycles of denaturation (94 °C for 35 s), annealing (60 °C for 30 s), extension (72 °C for 2 min) and a final extension at 72 °C for 5 min. PCR assays contained a positive control (template DNA of *F. verticillioides*) and a negative control with no DNA added. The amplification was performed in a T-100 thermal cycler (Bio Rad, Hercules, CA, USA). All PCR fragments were separated by electrophoresis by applying a tension of 110 V for about 1 h. Electrophoretic runs were visualized using an UV Image analyzer (Euroclone, Pero, Milan, Italy).

### 2.3. Determiantion of Fumonisin Biosynthesis by F. verticillioides In Vitro

#### 2.3.1. *F. verticillioides* Cultures

To determine in vitro fumonisin biosynthesis, cultures of *F. verticillioides* strains were obtained following the protocol indicated by Covarelli et al. [50] with slight modifications. In brief, 15 g of finely ground maize grains and 15 mL of deionized sterile water were added into 100 mL glass flasks (Duran, Mainz, Germany) to obtain the right moisture for allowing fungal development and then autoclaved three times at alternate days. Three flasks (replicates) for each *F. verticillioides* strain were then inoculated with a mycelium plug (0.6 cm diameter) taken from the growing edge of one-week-old pure fungal cultures of each strain developed on PDA at 22 °C in the dark. Three flasks (replicates) were used as controls by adding only a PDA plug. Flasks were incubated in the dark at 22 °C for 4 w, and developed cultures were then freeze-dried for 24 h using a lyophilizer instrument (Heto Powder Dry LL3000, Thermo Fisher Scientific, Waltham, MA, USA), ground with mortar and pestle and stored at −80 °C until further analysis.

#### 2.3.2. Fumonisin Extraction and LC-MS/MS Analysis

Each fungal culture was extracted and analyzed in triplicate according to the validated and routine procedure also described by Covarelli et al. [50] with slight modifications. In brief, 1 g of ground sample was extracted with 5 mL of methanol/water (75:25, *v*/*v*) following 60 min shaking. The extract was filtered through filter paper. Prior to liquid chromatography, tandem Mass Spectrometry (LC-MS/MS) analysis, the extract was diluted by default 1:50 with a mixture of methanol/water (60:40), then filtered through 0.45 µm syringe filter. Twenty microliters were injected into the LC-MS/MS apparatus. If fumonisin levels were out of the calibration range, a further dilution (1:500 or 1:5000) was applied to the raw extract and then re-analyzed.

LC-MS/MS analyses were performed on a QTrap MS/MS system, from Applied Biosystems (Foster City, CA, USA), equipped with an Electrospray Ionization (ESI) interface and a 1100 series micro-Liquid Chromatography system comprising a binary pump and a micro-autosampler from Agilent Technologies (Waldbronn, Germany). The analytical column was a Gemini^®^ C18 column (150 × 2 mm, 5 µm particles) (Phenomenex, Torrance, CA, USA), preceded by a Gemini^®^ C18 guard column (4 × 2 mm, 5 µm particles). The column oven was set at 40 °C. The flow rate of the mobile phase was 200 µL/min, and the injection volume was 20 µL.

The column effluent was directly transferred into the ESI interface, without splitting. Eluent A was water and eluent B was methanol, both containing 0.5% acetic acid. A gradient elution was performed as follows. The percentage of eluent B was increased from 40% to 80% in 10 min, kept constant 3 min, then increased to 100% in 1 min, and kept constant for 4 min. The column was re-equilibrated with 40% eluent B for 7 min. The ESI interface was used in positive ion mode with the following settings: temperature 350 °C; curtain gas, nitrogen, 30 psi; nebulizer gas, air, 10 psi; heater gas, air, 30 psi; ion spray voltage +4500 V. The mass spectrometer operated in Multiple Reaction Monitoring (MRM) mode. Mycotoxin quantification was performed by external calibration in neat solvent. The identity of fumonisins was confirmed by comparison with the analytical standard considering chromatography retention time and MRM transitions (ion ratios) in agreement with the official guidelines for mycotoxin identification by Mass Spectrometry [59]. Detection limits in maize fungal cultures were 0.002 µg/g for FB_1_ and 0.001 µg/g for FB_2_ and FB_3_.

Methanol (HPLC grade) and glacial acetic acid were purchased from Mallinckrodt Baker (Milan, Italy). Ultrapure water was produced by a Millipore Milli-Q system (Millipore, Bedford, MA, USA). Filter papers (Whatman no. 4) were obtained from Whatman International Ltd. (Maidstone, UK). HPLC syringe filters (regenerated cellulose, 0.45 µm) were from Alltech (Deerfield, IL, USA).

### 2.4. Genetic Structure of Different F. verticillioides Populations

For genetic diversity assessment, all *F. verticillioides* strains were cultured on PDA for 7 d. Mycelia were harvested, homogenized in liquid nitrogen, and genomic DNA was extracted using the method already described by Stępień et al. [60]. A pre-validated *FUM1*-specific marker that showed intraspecific polymorphism in *F. verticillioides* and *F. proliferatum* in previous studies [61,62] was used. Briefly, Fum1F1 (CACATCTGTGGGCGATCC)/Fum1R2 (ATATGGCCCCAGCTGCATA) primers were used for *FUM1* gene fragment PCR-based amplification and sequencing according to Waśkiewicz et al. [61]. Additionally, *FUM6*-*FUM7* and *FUM7*-*FUM8* intergenic regions were amplified using the primers Fum6eF (AGATTTCCCAACAGTGGCAG)/Fum7bR (GTTTGCTTGGTGGAACTGGT) and Fum7eF (ATCCGGTTGAGTTGGACAAG)/Fum8eR (GGAACAGATGCCCATACCAT) according to Stępień et al. [47].

The BigDye Terminator kit v. 3.1 (Life Technologies, Carlsbad, CA, USA) was used for fluorescent labeling according to the manufacturer’s instructions. DNA fragments were purified using alkaline phosphatase and exonuclease I (Thermo Fisher Scientific)) and precipitated using ice-cold 96% ethanol (Sigma Aldrich, St. Louis, MO, USA). Sequence reading was performed using Applied Biosystems equipment. Sequence reads were analyzed using BioEdit software [63] and aligned using MEGA5 software package [64] using Maximum Parsimony heuristics with standard settings. Based on *FUM1* sequences, the most parsimonious tree was calculated (bootstrap test with 1000 replications).

Sequences were compared to the NCBI GenBank-deposited sequence (*FUM* cluster NCBI (AF155733)) and, in addition, a total of five *F. verticillioides FUM1* sequences (F.v.F1.8.I.I, F.v.10I3 (*Pisum sativum*, Wiatrowo, Poland); F.v.KF3477, F.v.F1M1.1 (*Z. mays*, Poland); F.v.KF3537 (*Ananas comosus*, Costa Rica)) were used as references. A total of four *Fusarium proliferatum FUM1* sequences (15 *F. proliferatum* (*Z. mays*, Iran); *F. proliferatum* Gar3.2, Gar1 and Gar3.0 (*Allium sativum*, Poznan, Poland)) were used as outgroup.

### 2.5. Statistical Analysis

To analyze the in vitro fumonisin biosynthesis within each country of origin, total fumonisin content was submitted to ANOVA by allowing a different standard deviation per strain to comply with heteroscedasticity. Generalized least-squares were used for model fitting, as implemented in the gls() function of the nlme package [65] within the R statistical environment [66]. Heteroscedastic Welch’s *t*-tests were used for pairwise comparisons of strains, within country [67].

## 3. Results

### 3.1. Identity Confirmation of F. verticillioides

DNA extracted from the 90 *F. verticillioides* strains was subject to PCR assays using the species-specific primer pair VERT1/VERT2. As expected, a single fragment of 800 bp amplified in all the samples, thus confirming their identity as *F. verticillioides*.

### 3.2. Fumonisin Biosynthesis by F. verticillioides In Vitro

Data on the in vitro biosynthesis of FB_1_, FB_2_ and FB_3_ with the calculation of total fumonisins (sum of FB_1_, FB_2_ and FB_3_) by the 90 *F. verticillioides* strains are summarized in Table 1.

In general, this analysis revealed that 80% (*n* = 71) of the *F. verticillioides* strains investigated in this study were able to produce fumonisins at variable levels, while the remaining 20% (*n* = 19) showed undetectable levels (not detected; nd) of fumonisins and were considered, in this experimental condition, as non-producing strains.

Total fumonisins biosynthesized by all positive strains (*n* = 71) varied from 0.03 to 69.84 μg/g (average 7.88 μg/g), with FB_1_ being the most abundant analogue followed by FB_2_ and FB_3_. All positive strains (100%, *n* = 71) produced FB_1_ in levels ranging from 0.03–56.12 μg/g (average 5.9 μg/g), while 64 of 71 strains (90%) produced FB_2_ in levels ranging from 0.03–10.67 μg/g (average 1.6 μg/g). Finally, 59 of 71 strains (83%) biosynthesized FB_3_ in a range from 0.01–4.23 μg/g (average 0.7 μg/g). The average ratios of FB_1_:total fumonisins, FB_2_:total fumonisins and FB_3_:total fumonisins were 0.77, 0.13 and 0.05, respectively. The three fumonisin analogues analyzed in this study (FB_1_, FB_2_ and FB_3_) were simultaneously produced by 81% of positive strains (*n* = 58), while two analogues, FB_1_ and FB_2_ as well as FB_1_ and FB_3_, were simultaneously biosynthesized by 7% (*n* = 5) and 1% (*n* = 1) of positive strains, respectively. Finally, 7 out of 71 strains (10%) producerd only FB_1_. No strains biosynthesized FB_2_ or FB_3_ only.

In most cases, considering all producing strains (*n* = 71), differences in fumonisin production were detected among the strains isolated in the same country.

**Figure 2:**
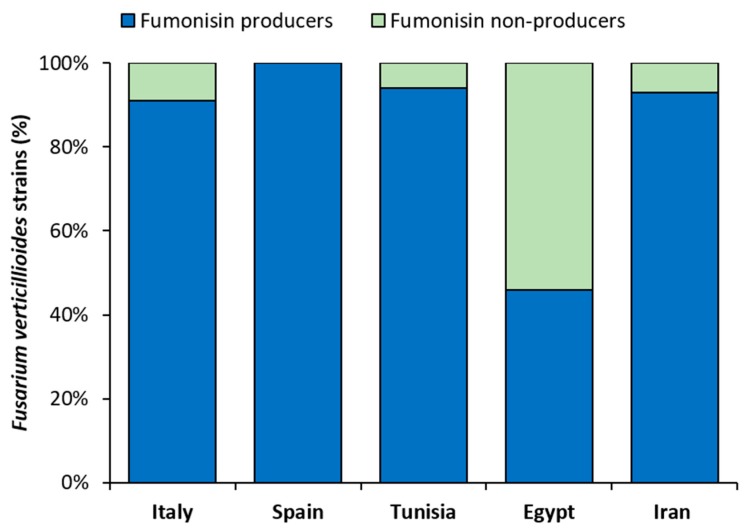
*Fusarium verticillioides* strains (%) isolated from maize kernels harvested in five Mediterranean countries that showed in vitro production of detectable (fumonisin producers) and non-detectable levels (fumonisin non-producers) of total fumonisins. Italy, *n* = 22; Spain, *n* = 9; Tunisia, *n* = 16; Egypt, *n* = 28; Iran, *n* = 15.

In detail, 20 out of 22 strains (91%; Figure 2) isolated from maize grains in Italy and analyzed in this study showed the ability to biosynthesize fumonisins in variable levels (Table 1). Total fumonisins biosynthesized by the Italian positive strains (*n* = 20) varied from 0.03 to 33.73 μg/g (average 9.98 μg/g). All fumonisin-producing Italian strains (100%, *n* = 20) biosynthesized FB_1_ in levels ranging from 0.03–23.87 μg/g (average 5.7 μg/g), while 19 out of 20 strains (95%) produced FB_2_ and FB_3_ in levels ranging from 0.03–5.63 μg/g (average 2.20 μg/g) and 0.05–4.23 μg/g (average 0.94 μg/g), respectively. The average ratios of FB_1_:total fumonisins, FB_2_:total fumonisins and FB_3_:total fumonisins were 0.71, 0.18 and 0.10, respectively. The three fumonisin analogues (FB_1_, FB_2_ and FB_3_) were simultaneously produced by 95% of positive Italian strains (*n* = 20), while 1 out of 20 strains (5%) produced only FB_1_. Strains ITEM 10027 and PG 36B showed a significantly higher biosynthesis of total fumonisins with respect to the other Italian strains (*p* < 0.02), with the exception of strains PG 58A1, PG 35A and PG 76A1 (*p* > 0.07).

Considering the Spanish strains analyzed in this study, all of them (100%, *n* = 9; Figure 2) were able to in vitro biosynthesize different levels of fumonisins. Total fumonisins produced by these strains ranged from 0.24 to 69.84 μg/g (average 14.01 μg/g) with FB_1_ being the most abundant (range 0.24–56.12 μg/g; average 10.9 μg/g), followed by FB_2_ (range 0.03–10.67 μg/g; average 2.4 μg/g) and FB_3_ (range 0.01–3.04 μg/g; average 0.7 μg/g). The average ratios of FB_1_:total fumonisins, FB_2_:total fumonisins and FB_3_:total fumonisins were 0.81, 0.15 and 0.04, respectively. Eight out of 9 strains (89%) simultaneously biosynthesized all three fumonisin analogues, while in 1 out of 9 strains (11%) only FB_1_ was detected. Strain 0-C-1–3 2/2 showed a significantly higher (*p* < 0.008) production of total fumonisins with respect to the other Spanish strains analyzed in this study.

Focusing on the Tunisian strains analyzed in this study, 15 out of 16 strains (94%; Figure 2) produced detectable amounts of fumonisins *in vitro*. Total fumonisin levels ranged from 0.33 to 13.59 μg/g, with an average production equal to 5.36 μg/g. Twelve out of 15 strains (80%) biosynthesized all the analogues, while 2 out of 15 strains (13%) produced FB_1_ and FB_2_, and the remaining strain (7%; *n* = 1) produced FB_1_ and FB_3_. The gradient of production did not differ from that detected for the other strains: FB_1_ (average 4.01 μg/g) > FB_2_ (average 0.86 μg/g) > FB_3_ (average 0.54 μg/g). The average ratios of FB_1_:total fumonisins, FB_2_:total fumonisins and FB_3_:total fumonisins were 0.76, 0.13 and 0.11, respectively. Strains M10, M14 and M1 showed significantly higher total fumonisin biosynthesis with respect to the other Tunisian strains (*p* < 0.02), with the exception of strains M21, M22, M7 and M8 (*p* > 0.05).

The *F. verticillioides* population isolated from maize kernels in Egypt and analyzed in this study showed a low percentage of fumonisin-producing strains (46%, *n* = 13; Figure 2) with an average total fumonisin production of 3.98 μg/g (range 0.22–11.23 μg/g). All producing strains biosynthesized FB_1_ (range 0.22–7.52 μg/g; average 2.95 μg/g), while 12 out of 13 strains (92%; average 0.77 μg/g) and 10 out of 13 strains (77%; average 0.40 μg/g) showed the ability to biosynthesize FB_2_ and FB_3_, respectively. In other words, 77% of producing strains were able to simultaneously produce all three fumonisin analogues, while 15% (*n* = 2) and 8% (*n* = 1) of the Egyptian strains showed the ability to biosynthesize FB_1_ and FB_2_ or FB_1_ alone, respectively. The average ratios of FB_1_: total fumonisins, FB_2_:total fumonisins and FB_3_:total fumonisins were 0.76, 0.17 and 0.09, respectively. The Egyptian strain F3 showed a significantly higher (*p* < 0.01) production of total fumonisins than F39, F29, F8, F4, F28, F9 and F32 strains.

In the *F. verticillioides* population isolated from maize kernels in Iran and anlyized in this study, a total of 14 fumonisin-producing strains were recovered (93%; Figure 2). Total fumonisins biosynthesized by all positive strains (*n* = 14) varied from 0.03 to 39.79 μg/g (average 7.28 μg/g). All producing Iranian strains (100%, *n* = 14) biosynthesized FB_1_ in levels ranging from 0.03–30.81 μg/g (average 5.57 μg/g), while 11 out of 14 strains (71%) produced FB_2_ in levels ranging from 0.1–7.23 μg/g (average 0.70 μg/g), and 10 out of 14 strains (64%) biosynthesized FB_3_ in levels ranging from 0.09–1.75 μg/g (average 0.70 μg/g), respectively. The average ratios of FB_1_:total fumonisins, FB_2_:total fumonisins and FB_3_:total fumonisins were 0.83, 0.14 and 0.07, respectively. The three fumonisin analogues (FB_1_, FB_2_ and FB_3_) were simultaneously produced by 64% of positive Iranian strains (*n* = 9), while 4 out of 14 strains (29%) produced only FB_1_, and 1 out of 14 strains (7%) biosynthesized FB_1_ and FB_2_. The Iranian strain 89 showed a significantly higher total fumonisin biosynthesis than the other strains from the same country (*p* < 0.01), with the exception of strains 5 and 7 (*p* > 0.05).

**Figure 3:**
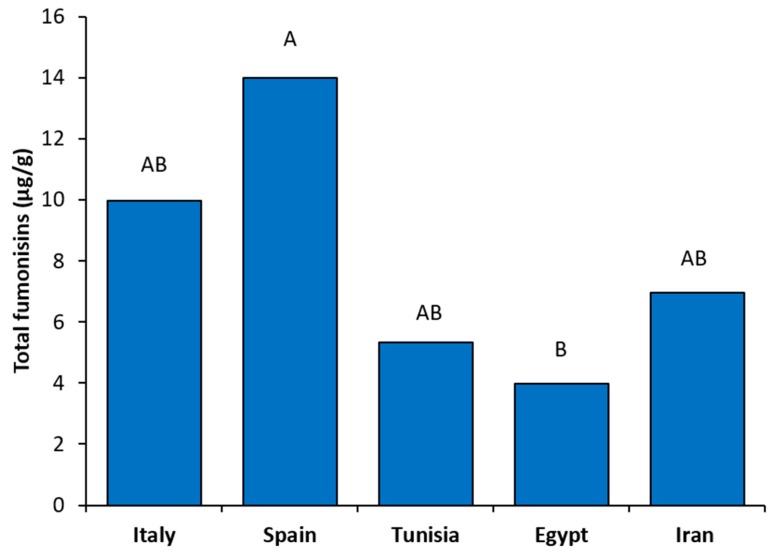
Average of total fumonisins (μg/g) biosynthesized by *Fusarium verticillioides* fumonisin-producing strains isolated from maize kernels harvested in each of the five countries analyzed in this study. Means with different letters are significantly different (*p* < 0.05).

Taking into account all fumonisin-producing strains of each country analyzed in this study, differences in total fumonisin biosynthesis among countries were also detected (Figure 3). In particular, the Spanish strains used in this study showed a significantly higher total fumonisin production (average 14.01 μg/g) than the Egyptian ones (average 3.98 μg/g) (*p* = 0.02). Also, the total fumonisin productions detected for the Italian (average 9.98 μg/g), Tunisian (average 5.36 μg/g) and Iranian (average 6.79 μg/g) strains were higher than the Egyptian ones and lower than the Spanish ones, even if no significant differences were recorded (*p* > 0.46 and *p* > 0.47, respectively) (Figure 3).

### 3.3. Genetic Structure and Variability of F. verticillioides Populations

We sequenced a portion of a divergent *FUM1* gene to evaluate the diversity among the five populations of *F. verticillioides* originating from various countries. All strains amplified DNA fragments of about 1100 bp in length. Additionally, the *FUM6*-*FUM7* (ca. 550 bp) and *FUM7*-*FUM8* (ca. 500 bp) intergenic regions were sequenced using the primers described previously [47].

The sequences were aligned, the ends trimmed manually using MEGA 5 software, and dendrograms of similarities were calculated. Interestingly, the intergenic regions did not show polymorphisms, which was rather unexpected, since these regions normally accumulated more point mutations than the coding regions. However, this means that the *F. verticillioides* strains characterized in this study, even if originating from different countries, were basically uniform (results not shown).

**Figure 4:**
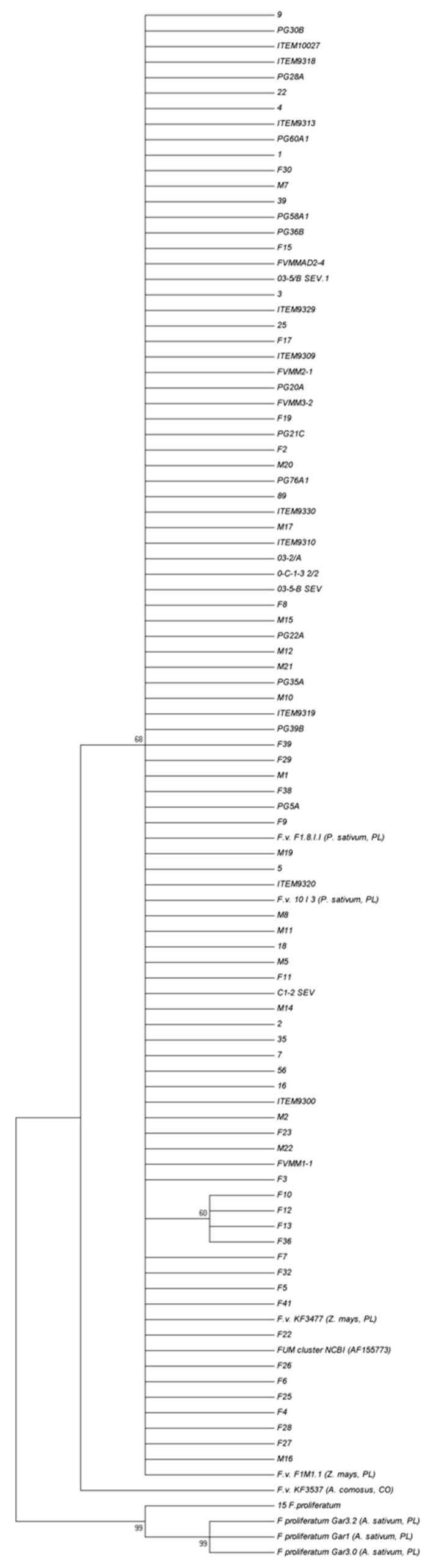
A most parsimonious tree calculated based on the partial *FUM1* sequences of 90 *Fusarium verticillioides* strains isolated from *Zea mays* of different origins using the maximum parsimony setting, bootstrap set to 50%, and 1000 replications were done. Five reference strains isolated from *Pisum sativum* (F.v. F1.8.I.I; F.v. 10 I 3), *Z. mays* (F.v. KF3477; F.v. F1M1.1) and *Ananas comosus* (F.v. KF3537) were added to the analysis, as well as the NCBI GenBank-deposited *FUM* cluster sequences (AF155773). Four *Fusarium proliferatum* sequences were also included as outgroup (15; Gar3.2; Gar1; Gar3.0).

Therefore, only slightly more polymorphic *FUM1* sequences were analyzed and shown (Figure 4). Apparently, neither geographical origin nor fumonisin production ability were correlated to the genetic diversity of the strain set, as almost all of them grouped together. Only four strains from Egypt (F10, F12, F13 and F36) were distinguished from the remaining strains at a bootstrap value of 60, including our five reference sequences [61] and NCBI GenBank-deposited *FUM* cluster sequences (AF155773) reported by Proctor et al. [45].

## 4. Discussion

This study was aimed at investigating the different ability of selected *F. verticillioides* strains isolated from maize kernels harvested in five Mediterranean countries to in vitro biosynthesize fumonisins as well as at characterizing their genetic structure to assess possible variabilities among them. So far, various studies have been conducted to analyze the ability of different *F. verticillioides* strains from diverse geographic areas to biosynthesize fumonisins. In several investigations, a large percentage of strains able to produce detectable amounts of these mycotoxins were usually found. However, the presence of strains that were not able to biosynthesize measurable levels of fumonisins was also reported. In this research, the majority of the strains isolated from maize grains in Italy, Spain, Tunisia and Iran, analyzed in this study, produced detectable levels of fumonisins (91%, 100%, 94% and 94% respectively; Figure 2), while the remaining part showed a lack of ability to produce measurable amounts of these mycotoxins. Similar percentages of fumonisin-producing strains (> 80%) were also detected in other *F. verticillioides* populations isolated from maize in Croatia [68], Spain [15,69], Italy [50], Iran [22], Egypt [17], Brazil [41,44,49], Korea [70], USA [71], Argentina [55,72] and from durum wheat in Argentina [2].

Conversely, in this study, only 46% of the analyzed Egyptian strains showed the ability to biosynthesize detectable amounts of fumonisins (Figure 2). Similarly to other studies, low incidences of producing strains were also recorded in other *F. verticillioides* populations such as those isolated from maize in Croatia (55%) [73], Taiwan (66%) [74] and Spain (36%) [14].

In general, the producing strains analyzed in this study biosynthesized fumonisin analogues following the “typical” gradient: FB_1_ > FB_2_ > FB_3_. A predominance of FB_1_ compared to the other analyzed fumonisin analogues was recovered also in other *F. verticillioides* populations such as those isolated from maize in Spain [15,75], Italy [76], Iran [22], Brazil [44,49], Argentina [55,72], Egypt [17], South Korea and South Africa [39]. In this study, no *F. verticillioides* strains producing more FB_2_ or FB_3_ than FB_1_ were recorded. Conversely, these types of strains were observed in *F. verticillioides* populations isolated from durum wheat in Argentina [2] and from maize and sorghum cultivated in the United States [77].

As known, fumonisin production within the *F. verticillioides* species could quantitatively vary due to the different biosynthetic ability of the different strains [24,40]. Also in this study, variability of fumonisin production among strains isolated in the same country was found, highlighting that mycotoxigenic diversity occurred within the five investigated *F. verticillioides* populations. Variability among *F. verticillioides* strains isolated from maize in the same country was commonly detected in many surveys in other parts of the world [2,8,15,17,22,44,49,55,73,74,75].

Variability in fumonisin production was also recorded among *F. verticillioides* strains isolated from different countries [30,39,71]. Also in this study, differences in fumonisin production among strains of different geographic origin were detected. In particular, the Spanish and Egyptian strains analyzed in this study showed a high level of mycotoxigenic variability, being the populations with the highest and the lowest fumonisin productions, respectively.

Interestingly, these two populations were also those with the highest and lowest percentages of fumonisin-producing (Spain) and non-producing (Egypt) strains. Conversely, the other three investigated populations of *F. verticillioides* (isolated from Italy, Tunisia and Iran) considered in this study did not show a significant variability of fumonisin production. In agreement with the results of Vogelgsang et al. [78], it is important to consider that in vitro results cannot be fully extrapolated to in vivo conditions because there are several factors influencing *Fusarium* infections and secondary metabolite production in the field. However, in vitro results could provide important information, which may be useful to understand intra-population variability within a single country as well as inter-population variability among different countries.

In this study, the mycotoxigenic characterization of *F. verticillioides* strains from different geographic origins was coupled to the study of the genetic structure of these populations. The genetic diversity of *F. verticillioides* has been studied using multiple techniques, including AFLP and RAPD methods [50,53,79]. Recently, however, direct sequencing of specific genomic regions has become more popular because of its high discrimination power and accuracy. The *FUM1* gene has already beeeen proven to be useful to assess species diversity inside the FFSC, serving as a source of phylogenetic and chemotypic markers [47], showing often higher levels of polymorphisms than constitutively expressed genes [e.g., *beta tubulin* (*tub2*) or *translation elongation factor 1α* (*tef-1α*)].

Our previous studies suggested there might be high levels of intraspecific genetic uniformity inside *F. verticillioides* populations, particularly when compared to the high diversity of the closely related species *F. proliferatum* [61,62,80,81]. The use of the *FUM1* gene sequence analysis allowed for discrimination of subpopulations likely related to the host species of origin. We assumed that a similar rule would be valid for *F. verticillioides*; therefore, we added some pea- and pineapple-derived strains to the analysis (Figure 4). It was also possible that geographical differences between populations would become visible.

However, in the present study we could not confirm this hypothesis. In fact, this was in accordance to previous findings, which did not reveal significant differences between *F. verticillioides* strains from different hosts [61]. This was also confirmed by the sequence analysis of the intergenic regions between *FUM6* and *FUM7* as well as *FUM7* and *FUM8* genes (results not shown), which were previously used for polymorphism screening [47]. The most likely explanation for this situation may be the endophytic type of growth observed for this pathogen in maize, which combined with the extensive seed material transfer between countries and continents made the population uniform across the world. Another possibility is that *FUM* cluster integrity and structure undergoes much more strict selection pressure in *F. verticillioides* than in *F. proliferatum*. This may implicate that fumonisin production by *F. verticillioides* is more essential to complete its life cycle than it is for *F. proliferatum*. This issue was already reported by Glenn et al. [82] but never confirmed for *F. proliferatum*.

The only outlier obtained in this study was a group of four strains (F10, F12, F13 and F36) isolated from Egypt (Figure 4), which was distinct from the remaining strains. Only one of these strains (F13) produced fumonisins in detectable amounts (Table 1). They should be further studied to explain their genetic diversity.

## 5. Conclusions

In this study, we analyzed fumonisin production as well as genetic structures of five *F. verticillioides* populations isolated from maize kernels in five Mediterranean countries.

The characterization of a selected number of strains per country does not allow a general conclusion to be drawn at the country level; however, the results obtained in these experimental conditions highlighted:

(i) the presence of an Egyptian population which differed from the others for its low percentage of fumonisin-producing strains;
(ii) the presence of significant differences in fumonisin production within the strains isolated in each of the surveyed countries and, in some cases, also among populations isolated from different countries;
(iii) the high level of genetic uniformity inside the populations analyzed;
(iv) the general absence of correlation between geographical origin and/or fumonisin production ability with the genetic diversity of the strain set;
(v) the presence of four Egyptian strains that were distinguished from the other strains at a bootstrap value of 60.

## Figures and Tables

**Table 1:** Strain ID, country of origin and fumonisin B_1_, fumonisin B_2_ and fumonisin B_3_ production (μg/g) with standard errors (±SE) by *Fusarium verticillioides* strains isolated from maize kernels harvested in five Mediterranean countries and analyzed in this study.

Strain ID	Origin	Fumonisin Production (μg/g) *								
		Fumonisin B_1_		Fumonisin B_2_		Fumonisin B_3_		Total Fumonisins **^,§^		
PG 21C	Italy	nd ^†^	-	nd	-	nd	-	nd	-	-
PG 39B	Italy	nd	-	nd	-	nd	-	nd	-	-
ITEM 9313	Italy	0.03	(±0.01)	nd	-	nd	-	0.03	(±0.01)	a
ITEM 9319	Italy	0.16	(±0.08)	0.03	(±0.01)	0.05	(±0.02)	0.24	(±0.11)	ab
PG 60A1	Italy	0.20	(±0.02)	0.04	(±0.01)	0.05	(±0.01)	0.29	(±0.02)	b
ITEM 9330	Italy	0.30	(±0.08)	0.05	(±0.01)	0.06	(±0.01)	0.41	(±0.09)	ab
ITEM 9320	Italy	0.63	(±0.60)	0.10	(±0.10)	0.08	(±0.07)	0.81	(±0.77)	ab
ITEM 9300	Italy	0.65	(±0.37)	0.11	(±0.06)	0.11	(±0.05)	0.87	(±0.48)	ab
PG 28A	Italy	1.01	(±0.40)	0.22	(±0.10)	0.25	(±0.08)	1.49	(±0.58)	ab
ITEM 9318	Italy	1.03	(±0.68)	0.22	(±0.15)	0.35	(±0.24)	1.59	(±1.07)	ab
PG 22A	Italy	1.67	(±1.52)	0.24	(±0.23)	0.25	(±0.22)	2.16	(±1.97)	ab
PG 20A	Italy	2.81	(±1.50)	0.66	(±0.35)	0.40	(±0.16)	3.87	(±2)	abc
ITEM 9310	Italy	6.56	(±3.09)	2.46	(±1.19)	0.68	(±0.29)	9.69	(±4.56)	abcd
PG 5A	Italy	6.99	(±0.89)	2.35	(±0.37)	0.85	(±0.06)	10.19	(±1.27)	cd
ITEM 9309	Italy	7.70	(±3.45)	2.23	(±1)	0.80	(±0.30)	10.74	(±4.74)	abcd
PG 76A1	Italy	8.78	(±4.50)	2.32	(±1.29)	1.24	(±0.60)	12.34	(±6.39)	abcde
PG 30B	Italy	10.36	(±1.25)	2.95	(±0.45)	1.26	(±0.26)	14.57	(±1.92)	d
ITEM 9329	Italy	10.71	(±2.32)	3.04	(±0.71)	0.84	(±0.16)	14.59	(±3.16)	cd
PG 35A	Italy	13.30	(±6.96)	4.39	(±2.26)	1.78	(±0.80)	19.47	(±10)	abcde
PG 58A1	Italy	19.39	(±5.28)	7.51	(±1.73)	2.16	(±0.15)	29.07	(±7.05)	abcde
ITEM 10027	Italy	23.64	(±1.57)	7.22	(±0.44)	2.49	(±0.05)	33.35	(±1.99)	e
PG 36B	Italy	23.87	(±0.44)	5.63	(±1.56)	4.23	(±0.19)	33.73	(±1.49)	e
03-2/A	Spain	0.24	(±0.17)	nd	-	nd	-	0.24	(±0.17)	
FVMM 3-2	Spain	0.78	(±0.29)	0.03	(±0.03)	0.01	(±0.01)	0.82	(±0.33)	a
C1-2 SEV	Spain	2.24	(±1.19)	0.53	(±0.42)	0.01	-	2.77	(±1.61)	ab
FVMM 2-1	Spain	2.60	(±1.60)	0.55	(±0.46)	0.24	(±0.13)	3.38	(±2.17)	ab
FVMM AD 2-4	Spain	6.38	(±3.28)	1.61	(±0.91)	0.20	(±0.05)	8.19	(±4.19)	ab
03-5/B SEV.1	Spain	6.63	(±1.08)	1.31	(±0.31)	0.31	(±0.05)	8.24	(±1.43)	b
03-5/B SEV	Spain	7.70	(±3.57)	1.81	(±0.92)	1.06	(±0.66)	10.57	(±5.01)	ab
FVMM 1-1	Spain	15.63	(±4.19)	4.68	(±1.25)	1.77	(±0.33)	22.08	(±5.74)	ab
0-C-1-3 2/2	Spain	56.12	(±5.31)	10.67	(±1.35)	3.04	(±0.21)	69.84	(±6.57)	c
M16	Tunisia	nd	-	nd	-	nd	-	nd	-	-
M11	Tunisia	0.29	(±0.07)	0.04	(±0.02)	nd	-	0.33	(±0.09)	a
M19	Tunisia	0.30	(±0.07)	0.03	(±0.02)	0.11	(±0.03)	0.45	(±0.11)	a
M12	Tunisia	0.56	(±0.23)	0.12	(±0.05)	0.06	(±0.02)	0.74	(±0.30)	ab
M15	Tunisia	0.47	(±0.17)	0.06	(±0.02)	0.27	(±0.08)	0.80	(±0.28)	ab
M20	Tunisia	0.92	(±0.13)	nd	-	0.01	-	0.93	(±0.13)	b
M17	Tunisia	0.91	(±0.21)	0.12	(±0.03)	0.55	(±0.12)	1.58	(±0.36)	ab
M5	Tunisia	2.55	(±1.43)	0.27	(±0.26)	nd	-	2.83	(±1.69)	ab
M2	Tunisia	3.21	(±1.32)	0.61	(±0.31)	0.01	-	3.82	(±1.63)	ab
M8	Tunisia	3.53	(±1.80)	1.01	(±0.58)	0.01	-	4.55	(±2.39)	abc
M7	Tunisia	3.80	(±3.05)	0.77	(±0.75)	0.40	(±0.32)	4.97	(±4.11)	abc
M22	Tunisia	6.85	(±3.59)	1.15	(±0.40)	2.07	(±0.47)	10.07	(±4.45)	abc
M21	Tunisia	7.10	(±4.93)	1.47	(±1.24)	1.72	(±1.09)	10.29	(±7.24)	abc
M1	Tunisia	8.82	(±1.28)	2.16	(±0.35)	0.68	(±0.23)	11.66	(±1.81)	c
M14	Tunisia	10.50	(±0.10)	1.72	(±0.12)	1.07	(±0.10)	13.28	(±0.18)	c
M10	Tunisia	11.07	(±1.71)	2.48	(±0.55)	0.04	(±0.03)	13.59	(±2.23)	c
F2	Egypt	nd	-	nd	-	nd	-	nd	-	-
F6	Egypt	nd	-	nd	-	nd	-	nd	-	-
F7	Egypt	nd	-	nd	-	nd	-	nd	-	-
F10	Egypt	nd	-	nd	-	nd	-	nd	-	-
F12	Egypt	nd	-	nd	-	nd	-	nd	-	-
F19	Egypt	nd	-	nd	-	nd	-	nd	-	-
F22	Egypt	nd	-	nd	-	nd	-	nd	-	-
F23	Egypt	nd	-	nd	-	nd	-	nd	-	-
F25	Egypt	nd	-	nd	-	nd	-	nd	-	-
F26	Egypt	nd	-	nd	-	nd	-	nd	-	-
F27	Egypt	nd	-	nd	-	nd	-	nd	-	-
F30	Egypt	nd	-	nd	-	nd	-	nd	-	-
F36	Egypt	nd	-	nd	-	nd	-	nd	-	-
F38	Egypt	nd	-	nd	-	nd	-	nd	-	-
F41	Egypt	nd	-	nd	-	nd	-	nd	-	-
F39	Egypt	0.22	(±0.02)	nd	-	nd	-	0.22	(±0.02)	a
F29	Egypt	0.81	(±0.05)	0.19	(±0.04)	0.12	(±0.03)	1.12	(±0.11)	b
F8	Egypt	0.96	(±0.90)	0.34	(±0.33)	nd	-	1.29	(±1.23)	ab
F4	Egypt	1.18	(±0.08)	0.10	(±0.02)	0.08	-	1.35	(±0.11)	b
F28	Egypt	1.08	(±0.69)	0.21	(±0.13)	0.09	(±0.05)	1.38	(±0.87)	ab
F9	Egypt	1.14	(±0.79)	0.15	(±0.13)	0.32	(±0.25)	1.61	(±1.17)	ab
F32	Egypt	1.11	(±0.34)	0.72	(±0.27)	0.38	(±0.20)	2.21	(±0.80)	ab
F5	Egypt	4.10	(±2.16)	0.70	(±0.40)	0.05	(±0.03)	4.85	(±2.60)	abc
F11	Egypt	3.56	(±1.88)	0.70	(±0.44)	0.58	(±0.37)	4.85	(±2.68)	abc
F17	Egypt	4.35	(±3.24)	2.03	(±1.57)	nd	-	6.38	(±4.81)	abc
F13	Egypt	6.02	(±1.45)	0.88	(±0.11)	0.33	(±0.12)	7.23	(±1.67)	abc
F15	Egypt	6.32	(±4.25)	1.29	(±0.98)	0.38	(±0.22)	7.99	(±5.45)	abc
F3	Egypt	7.52	(±0.08)	1.95	(±0.15)	1.75	(±0.15)	11.23	(±0.32)	c
35	Iran	nd	-	nd	-	nd	-	nd	-	-
4	Iran	0.03	(±0.02)	nd	-	nd	-	0.03	(±0.02)	a
25	Iran	0.10	(±0.02)	nd	-	nd	-	0.10	(±0.02)	b
2	Iran	0.27	(±0.08)	nd	-	nd	-	0.27	(±0.08)	ab
9	Iran	0.47	(±0.37)	nd	-	nd	-	0.47	(±0.37)	ab
18	Iran	1.21	(±0.25)	0.10	(±0.05)	0.09	(±0.04)	1.40	(±0.35)	abc
39	Iran	1.65	(±0.45)	0.19	(±0.18)	0.42	(±0.12)	2.26	(±0.73)	abc
56	Iran	2.21	(±1.12)	0.34	(±0.18)	0.30	(±0.16)	2.85	(±1.42)	abc
1	Iran	3.94	(±0.76)	0.56	(±0.18)	0.22	(±0.07)	4.72	(±1)	c
3	Iran	4.48	(±1.22)	0.76	(±0.22)	0.47	(±0.16)	5.71	(±1.59)	abc
22	Iran	4.61	(±1.38)	1.65	(±0.53)	nd	-	6.26	(±1.91)	abc
16	Iran	4.66	(±1.63)	1.48	(±0.58)	0.40	(±0.18)	6.55	(±2.39)	abc
5	Iran	9.92	(±5.52)	2.15	(±1.35)	1.17	(±0.71)	13.25	(±7.59)	abcd
7	Iran	13.65	(±4.74)	3.23	(±1.15)	1.45	(±0.50)	18.33	(±6.40)	abcd
89	Iran	30.81	(±4.39)	7.23	(±1.01)	1.75	(±0.28)	39.79	(±5.25)	d

* values represent the average (±SE) of three biological replicates. ** sum of fumonisin B_1_, fumonisin B_2_ and fumonisin B_3_. ^†^ nd: not detected (<0.002 μg/g for fumonisin B_1_ and <0.001 μg/g for fumonisin B_2_ and fumonisin B_3_). ^§^ within the same country of origin, means followed by different letters are significantly different (*p* < 0.05).
